# Cuspal Deflection and Temperature Rise of MOD Cavities Restored through the Bulk-Fill and Incremental Layering Techniques Using Flowable and Packable Bulk-Fill Composites

**DOI:** 10.3390/ma13245664

**Published:** 2020-12-11

**Authors:** Roberto De Santis, Vincenzo Lodato, Vito Gallicchio, Davide Prisco, Francesco Riccitiello, Sandro Rengo, Carlo Rengo

**Affiliations:** 1 Institute of Polymers, Composites and Biomaterials—National Research Council of Italy, V.le J.F. Kennedy 54—Mostra d’Oltremare Pad. 20, 80125 Naples, Italy; rosantis@unina.it; 2 Department of Neurosciences, Reproductive and Odontostomatological Sciences, University “Federico II” of Naples, via S. Pansini 5, 80131 Naples, Italy; vincenzo.lodato@unina.it (V.L.); d.prisco@unina.it (D.P.); riccitie@unina.it (F.R.); sanrengo@unina.it (S.R.); 3 Department of Prosthodontics and Dental Materials, University of Siena, 53100 Siena, Italy; carlorengo@alice.it

**Keywords:** cuspal deflection, composite material shrinkage, bulk-fill composite, mechanical properties, dental materials, temperature rise

## Abstract

*Background*: The aim of this study was to investigate cuspal deflection caused by material shrinkage and temperature rise occurring in the pulp chamber during photopolymerization. The aim of this study was also to investigate the effect of flowable and packable bulk-fill composites on cuspal deflection occurring in mesio-occlusal–distal (MOD) cavities restored through the bulk-fill or through the incremental layering technique. Additionally, mechanical and thermal properties of bulk-fill composites were considered. *Methods*: Two bulk-fill composites (high-viscosity and low-viscosity), largely differing in material composition, were used. These composites were characterized through linear shrinkage and compressive test. Cuspal deformation during restoration of mesio-occlusal–distal cavities of human premolars was evaluated using both the bulk-fill and the incremental layering techniques. Temperature rise was measured through thermocouples placed 1 mm below the cavity floor. *Results*: Shrinkage of the flowable composite was significantly higher (*p* < 0.05) than that of packable composite, while mechanical properties were significantly lower (*p* < 0.05). For cusp distance variation, no significant difference was observed in cavities restored through both restorative techniques, while temperature rise values spanned from 8.2 °C to 11.9 °C. *Conclusions*: No significant difference in cusp deflection between the two composites was observed according to both the restorative techniques. This result can be ascribed to the Young’s modulus suggesting that the packable composite is stiffer, while the flowable composite is more compliant, thus balancing the cusp distance variation. The light curing modality of 1000 mW/cm^2^ for 20 s can be considered thermally safe for the pulp chamber.

## 1. Introduction

In the last few decades, composite materials have been widely used for conservative dentistry, although resin-based composites are subjected to volumetric contraction during polymerization, providing satisfactory aesthetics. Due to this contraction, shrinkage stress can cause an adhesive debonding at the tooth–adhesive–composite interfaces [1,2]. Bacterial leakage, dentinal sensibility, and secondary caries are the clinical effects of interfacial microleakage [3,4]. Dental composite materials have been largely studied and developed to reduce shrinkage stress and avoid side effects by varying filler amount, monomer structure or chemistry, and polymerization reaction dynamics. A method to reduce material shrinkage is to delay the gel point, i.e., the point at which rheological properties vary due to the formation of a three-dimensional (3D) network. By not exceeding the gel point, the polymer chains formed are flexible and, consequently, the material has a low viscosity. During photopolymerization, before gelation, polymer networks start to form and viscosity increases. At this stage, shrinkage stress is compensated for by the plastic flow offered by the pre-gelation phase and by the achievement of stress relaxation. Once the gel point has been exceeded, polymerization contraction can no longer be compensated for by the material, thus determining the final stress achieved [5].

Many techniques have been developed in order to reduce the stress at the adhesive interface. The incremental restoration technique, in direct restorations, was developed to vary the shrinkage pattern [6]. This technique is based on filling the tooth cavity with microhybrid or nanohybrid conventional composite resin increments. On the other hand, the incremental technique increases both the clinical chair time required for dental reconstruction and the technique sensitivity [7,8].

The high translucency and a powerful initiator system confer to bulk-fill resin composites a high depth of cure [9,10]. Compared to traditional incremental filling techniques, cavities with a depth higher than 4 mm can be filled through the bulk-fill technique, thus reducing the chair time to fill a cavity [7,11]. Depending on rheological properties, bulk-fill RBCs (Resin Based Composites) can be classified as flowable and packable [12]. Flowable composites are low-viscosity RBCs directly injected into the cavity through a needle with the advantage of a self-adaption to the cavity walls. One of the first flowable bulk-fill materials on the market is represented by the Smart Dentin Replacement (SDR; Sirona Dentsply, Konstantz, Germany) bulk-fill composite. However, capping with a conventional composite material is recommended to avoid wear [8,13,14].

Instead, high-viscosity bulk-fill RBCs show mechanical properties similar to conventional nanohybrid and microhybrid composite materials. The application of these high-viscosity materials into the cavity is achieved through a traditional spatula or through a sonic vibration handpiece to reduce viscosity while injecting materials such as SonicFill 2 (SFL; Kerr Corporation, Orange, CA, USA) [9]. Due to the high percentage of resin matrix, bulk-fill flowable RBCs should achieve a high shrinkage. Several studies demonstrated that the lower shrinkage is achieved at the bottom of the cavity, because the light cure intensity decreases as the curing depth increases, thus generating a polymerization degree gradient through the composite [15,16,17]. A polymerization degree gradient through the whole bulk-fill composite thickness could involve a lower monomer-to-polymer conversion and a consequent genotoxicity release. Many studies regarding the correlation between the degree of conversion and the increase in toxicity have been conducted, and it was proven that bulk-fill composites have a high depth of cure with a high degree of conversion, thus indicating an adequate biocompatibility [18]. Compared to other bulk-fill resin composites for posterior teeth restoration, SonicFill 2 shows a higher inorganic filler amount [19]. Mechanical properties of SDR are higher than those of other flowable RBCs but lower than those of regular nanohybrid or microhybrid RBCs; as a consequence, SDR shows higher plastic deformation and creep than other low-viscosity RBCs. However, enamel wear by antagonistic teeth restored with dental composite has been recognized [8,20]. For this reason, clinicians recommend capping with 2 mm occlusal increments using conventional high-viscosity composite [21]. It has been reported that SonicFill 2 contracts less than SDR, and the volumetric shrinkage values compared with other flowable materials are significantly different. The different amount of organic matrix in the material has a direct relationship with the shrinkage values. SonicFill 2 is a composite material with a very high filler content and, thus, it is expected to have a lower shrinkage value than a flowable material which has a higher resin content. SonicFill 2 achieves the properties of a hybrid material after polymerization, but it has a consistency similar to that of a flowable material when it is placed through a sonic vibration handpiece. SDR has a higher volumetric shrinkage, but stress can be lower than Sonicfill 2. This can be attributed to the new modulator that slows the polymerization rate, reducing the stress [22]. The difference in volumetric shrinkage, due to the viscosity of the material, was demonstrated by Kim et al. Their study proved that a higher shrinkage and a lower modulus are achieved by a low-viscosity composite (e.g., SDR) compared to a high-viscosity composite (e.g., SonicFill 2) [21]. Sung-Ae et al. showed that bulk-fill composites with low filler content have a higher polymerization shrinkage than those with high filler content [23]. An important aspect related to the filler content is the elastic modulus of the composite material. Ilie et al. tested the mechanical properties of different resin-based composite materials. Their study resulted in a variation in the elastic modulus due to a variation of the filler content. In particular, SonicFill 2 had a filler loading higher than SDR, thus achieving a significantly higher value of the elastic modulus [8]. This result is in accordance with other studies in which SonicFill 2 achieved significantly greater values of flexural and compressive strength compared to SDR, thanks to the filler loading [24]. Other studies demonstrated that SDR has a lower flexural and compressive strength compared with other bulk-fill composites [25].

Clinically, a main concern of bulk-fill RBCs is a potentially increased shrinkage stress developing at the composite–tooth wall interface, as well as a temperature rise occurring for the polymerization of massive amount of composite material. Polymerization shrinkage can be directly evaluated through volumetric or linear measurements, based on 3D imaging or displacement transducers, respectively [26,27,28,29]. From a clinical point of view, an accurate method to evaluate the effects of composite shrinkage on dental tissues is the cuspal displacement measurement, through a measurement of the intercuspal distance variation with length. It is reported that teeth with small cavities deform less than those with large cavities, and the intercuspal distance decreases significantly in the first 60 min after starting curing. A gradual recovery of the cusps toward the original dimensions over a longer period has also been suggested [30]. Fleming et al. measured cuspal distance variation by measuring the buccal and lingual cuspal deflection of the extracted teeth through a twin displacement gauge sensor, and restoration was achieved through an incremental technique with eight increments. The filling technique minimized cuspal deflection by constraining the cusps, resulting in an underestimation of the deflection expected when one cusp was not constrained [31]. Kwaon et al. measured cuspal deflection through LVDT (Linear Variable Differential Transformer) probes using bulk-fill and incremental techniques. The incremental filling technique yielded significantly lower cuspal deflection than the bulk-filling technique [6]. A microscope with a micrometer stage was used by Alomari et al. to measure the distance between the cusp tips. The study resulted in high values of the cusps deflection, with a maximum deflection of 47 µm and a minimum of 23 µm. These high values have been ascribed to the strong bonding between the restoration and the cavity walls. A subsequent water immersion of the samples for 24 h resulted in a cusp relaxation between 12 μm and 17 μm [32].

Little is known on cuspal deflection as bulk-fill composites are used for restoring mesio-occlusal–distal (MOD) cavities of premolars according to the incremental or bulk-fill techniques. Kim et al. evaluated cuspal deflection of aluminum teeth according to a variety of bulk-fill composites considering both the incremental and the bulk-fill techniques [33]. The aim of this investigation was to evaluate cuspal deformation during restoration of MOD cavities of human premolars by using both the bulk-fill and the incremental layering techniques using two bulk-fill composite materials largely differing in viscosity.

The null hypothesis is that different layering techniques and different types of composite materials would not affect the cuspal distance variation and temperature rise.

## 2. Materials and Methods

Two composite materials, namely, SDR flow+ and SonicFill 2, were used. These composites were characterized according to linear shrinkage and compression tests, while dental cuspal deformation and temperature increase during restoration of MOD cavities of human premolars were investigated using both the incremental and the bulk-fill techniques.

### 2.1. Composite Materials

Two bulk-fill RBCs largely differing in material composition and rheological properties were tested. SDR flow+ represented one component as a light cured, radiopaque, flowable resin composite. SDR is available in pre-dosed (0.25 g) compula tips for intraoral application. Sonicfill 2 is a packable composite applied through a vibration handpiece, providing the sonic-activated dispensation of the composite material, thus reducing viscosity during placement [9].

The viscosity of SDR is expected to be similar to that of flowable restorative composites (viscosity lower than 1 kPa·s), while the viscosity of SFL is expected to be similar to that of highly filled RBCs (viscosity higher than 100 kPa·s) [12]. The polymeric matrix of the investigated composite materials was based on bisphenol A-glycidyl methacrylate (Bis-GMA), ethoxylated bisphenol A dimethacrylate (EBPADMA), urethane dimethacrylate (UDMA), and triethylene glycol dimethacrylate (TEGDMA). Table 1 depicts the material composition of the two bulk-fill RBCs and restoration techniques, and the initiator system of both composites was based on camphorquinone.

### 2.2. Composite Material Characterization

**Figure 1:**
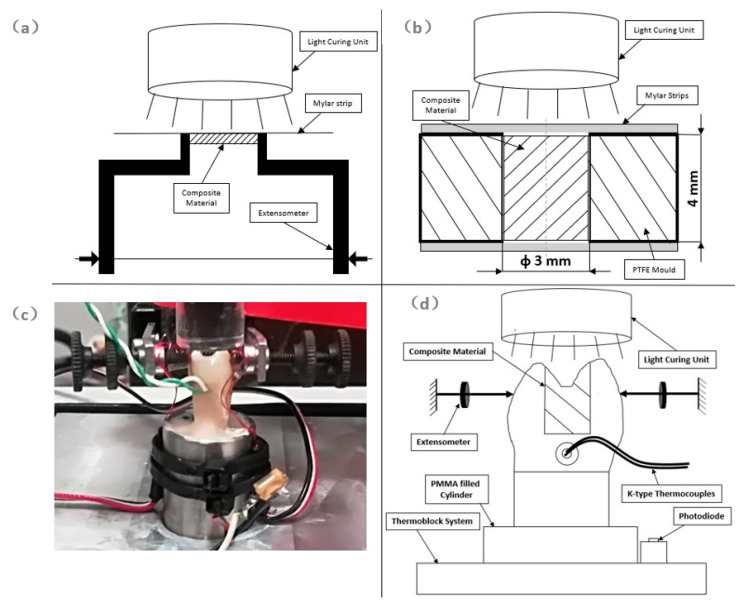
(**a**) Schematic of linear shrinkage evaluated in the direction perpendicular to the light curing front; (**b**) preparation of cylindrical specimens for compression test with diameter of 3 mm and height of 4 mm; (**c**) set-up adopted for the simultaneous measurement of cuspal distance, temperature, and light intensity; (**d**) description of the elements involved for the simultaneous measurement of cuspal distance, temperature, and light power.

Linear shrinkage (Figure 1a) of SFL and SDR bulk composites was investigated up to 3600 s through a protocol previously described [2]. Briefly, bulk composites were injected into prismatic PTFE (Polytetrafluoroethylene) molds of 5.0 mm × 5.0 mm × 1.5 mm. A Mylar strip was used to handle the composite and to position each specimen between the mechanical arms of an Instron extensometer 2620-601 (Instron Ltd., High Wycombe, UK). Shrinkage measurements were evaluated in the direction perpendicular to the light curing front. A filtered photocell obtained from a Demetron LED (Light Emitting Diode) radiometer (Kerr Corporation) was employed to monitor light power level. The light curing unit Swiss Master Light (EMS, Nyon, Switzerland) at an intensity level of 1000 mW/cm^2^ and exposure time of 10 s was used to light-cure composite materials. Light power and linear shrinkage data were simultaneously acquired at a speed of 50 p/s up to 3600 s using the National Instrument DAC (Data Acquisition and Control) (National Instruments, Austin, TX, USA) driven by Signal Express software (National Instruments). Five specimens for each bulk-fill composite were used, and data at 300 s and 3600 s were analyzed using two-way ANOVA followed by Tukey’s test at a critical value of 0.05.

Mechanical properties of SFL and SDR bulk-fill composites were investigated through compression tests (Figure 1b). Bulk-fill composites were injected into prismatic PTFE molds containing a cylindrical cavity having a 3 mm diameter and a 4 mm height. Mylar strips placed in the bottom and top sides of the cylindrical PTFE cavity were used to prevent material flow and oxidation of the composite material during the light curing process. The light curing unit Swiss Master Light (EMS) at an intensity level of 1000 mW/cm^2^ and exposure time of 20 s was used to light-cure composite materials. Specimens were kept in a dark environment at room temperature for 48 h before mechanical testing. An Instron 5566 (Instron Ltd.) testing machine, equipped with a 5 kN load cell, was employed for compression tests at a speed of 1 mm/min. The experimentally measured dynamometer compliance was 4.18 × 10^−5^ mm/N, and this value was considered to determine the true deformation of short specimens in compression. Mechanical strength was evaluated by considering the ratio of the maximum applied force and the cross-section area of the specimen, and strain to failure was calculated as the ratio of the maximum displacement and the specimen height, while Young’s modulus in compression was evaluated through the steepness of the stress–strain curve in the elastic region. Compression tests were performed within 1 hour (t0) or 72 h (t72h) of polymerization. Five replicates for each composite and for each time-point were used. Data were analyzed using two-way ANOVA followed by Tukey’s test at a critical value of 0.05.

### 2.3. Selection of Teeth

A total of 40 extracted upper premolars for orthodontic treatments were assigned to this study approved by the Ethics Committee of the University of Naples Federico II, with protocol number 137 2017. Before testing, caries- and defect-free premolars were selected. They were sterilized with HClO 2.5% solution, and then stored in distilled water until testing. Teeth were selected according to an average length of 22 ± 1 mm, a buccal–lingual dimension of 7 ± 1 mm, and a disto-mesial distance of 9 ± 1 mm. Teeth dimensions were measured with a digital caliper (Mitutoyo, Takatsuku, Japan). Teeth were fixed in a cylindrical metal mold of 16 mm diameter using acrylic resin. Each tooth was X-ray scanned with the Partner 70 equipment (Anthos, Bologna, Italy) in the mesial–distal, bucco-lingual, and occlusal–apical projections at 70 kV for 0.08 s. Dental radiographs were processed with the MicroDicom viewer v3.0.1 software (MicroDicom, Sofia, Bulgaria).

### 2.4. MOD Cavity Preparation

Standardized MOD cavities were prepared in each premolar with a 4 mm cavity depth and 3 mm intercuspidal width. Cavities were obtained through a diamond bur mounted on the turbine Fona8080 (Fonadental, Assago, Italy) on a high-speed contra-angle. Dimensions of each cavity preparation were measured using the digital caliper. Buccal and palatal walls of each cavity were prepared parallel to each other.

Teeth cusps and restorations MOD cavity dimensions are reported in Table 2.

Teeth were randomly divided into two groups (SDR and SFL). Samples of Group SDR were restored with SDR flow+ (Sirona Dentsply) composite, while samples of Group SFL were restored with SonicFill 2 (Kerr Corporation) composite. Each group was then divided into two subgroups (SFLB, SFLI and SDRB, SDRI) according to the restorative technique (Table 1). Groups SFLB and SDRB were restored using the bulk-fill technique (4 mm thickness), while groups SFLI and SDRI were restored through the incremental technique (two increments, 2 mm each).

### 2.5. Adhesive Protocol and Composite Restoration

After cavity preparation, teeth were subjected to an adhesive procedure: acid etching (enamel and dentine etching for 30 s and 15 s, respectively) using 37% phosphoric acid (Gerhò, Bolzano, Italy), rinsing and drying for 5 s, application of the adhesive system Optibond SE (Kerr Corporation), and photopolymerization with the Swiss Master Light (EMS) curing unit at an intensity of 1000 mW/cm^2^. SDR flow+ was injected into the cavity using a specific manual gun. SonicFill was dispensed directly into dental cavities using the Sonicfill handpiece.

### 2.6. Cuspal Distance Variation Measurements

Premolars were cemented in aluminium cylinders (D = 16 mm) using a low-temperature self-curing acrylic resin. Each sample was heated at 35 °C through a ThermoBlock system (Falc, Genova, Italy), and its temperature was kept constant during the test (Figure 1c,d).

The Instron Extensometer A1439-1014 (Instron Ltd.) was used to measure the variation in the distance between cusps during photopolymerization and during the dark reaction phase up to 3600 s. A filtered photocell, obtained from a Demetron LED radiometer (Kerr Corporation), was employed to monitor light power level. Disposable K-type thermocouples (RS components, Corby, UK), placed into a standardized hole created 1 mm below the cavity floor (Figure 1d), were used to measure temperature variation. The light curing unit Swiss Master Light (EMS) at an intensity level of 1000 mW/cm^2^ and exposure time of 20 s was employed to cure bulk-fill composites. Before each test, the PM100D ThorLab energy meter console (ThorLabs, Newton, NJ, USA), equipped with a S121C sensor (ThorLabs) and connected to the PMD100D software running under LabView (National Instruments), was employed to measure power output of the light curing unit.

Cuspal distance, temperature, and light power data were simultaneously acquired at a speed of 50 p/s up to 3600 s using the National Instrument DAC (National Instruments) driven by Signal Express software (National Instruments).

Five specimens for each bulk-fill composite and for each restorative technique (Table 1) were used, and data at 300 s and 3600 s were analyzed using two-way ANOVA followed by Tukey’s test at a critical value of 0.05.

This study was approved by the Ethics Committee of the University of Naples Federico II, with protocol number 137-2017.

### 2.7. Statistical Analysis

Data were analyzed using two-way ANOVA followed by Tukey’s test at a critical value of 0.05. Cuspal distance variation was considered as the dependent variable, while the composite material (i.e., SDR and SonicFill) and the restoration technique (i.e., bulk-fill and incremental layer) were considered as the independent variables. The product of the composite material and restoration technique provided the interaction effect of the type of composite and the restoration technique.

## 3. Results

**Figure 2:**
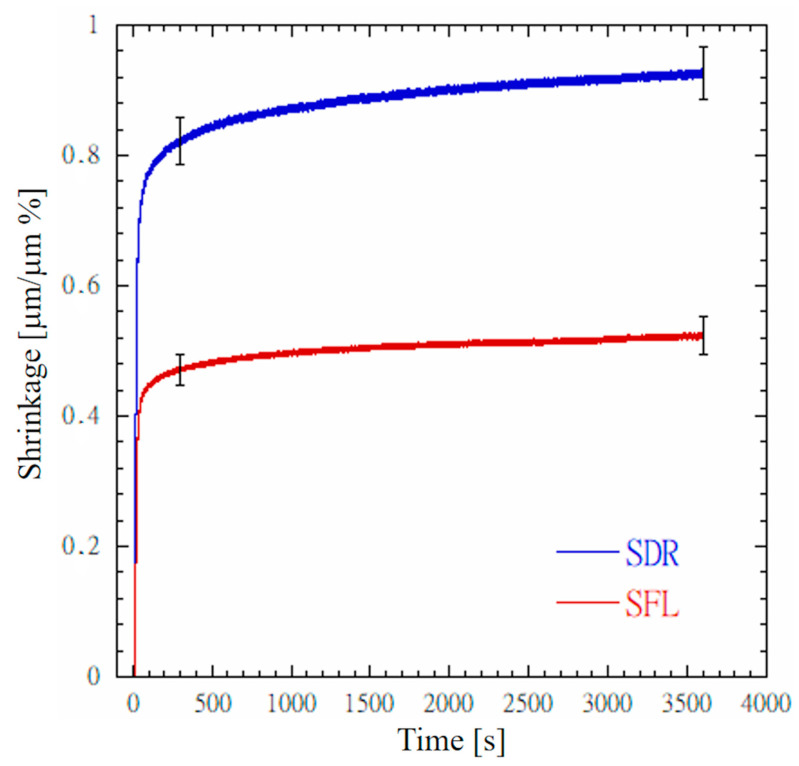
Shrinkage profiles recorded for SDR and SFL bulk-fill composites.

Figure 2 depicts typical shrinkage profiles recorded for SDR and SFL. A steep shrinkage profile can be observed for both composites as the light was turned on. After 300 s, the mean shrinkage value of SFL was significantly lower (*p* < 0.05) than that of SDR. Mean shrinkage values for SDR and SFL were 0.822 ± 0.037 µm/µm% and 0.471 ± 0.023 µm/µm%, respectively. As expected, shrinkage continued to increase during the dark reaction phase, and, at 3600 s, its values were significantly higher (*p* < 0.05) than those reported at 300 s. The mean shrinkage values at 3600 s for SDR and SFL were 0.925 ± 0.041 µm/µm% and 0.523 ± 0.029 µm/µm%, respectively.

Table 3 reports the mechanical properties measured in compression for SDR and SFL bulk-fill composites. At both time points, SFL showed a compressive strength (σ) significantly higher (*p* < 0.05) than that recorded for SDR. At both time points, the Young’s modulus (E) of SFL was also significantly higher (*p* < 0.05) than that recorded for SDR. Instead, at both time points, the strain to failure (ε) of SFL was significantly lower (*p* < 0.05) than that measured for SDR. The difference in mechanical properties of the two bulk-fill composites suggests that SFL is stiffer than SDR, but SDR is more compliant than SFL.

**Figure 3:**
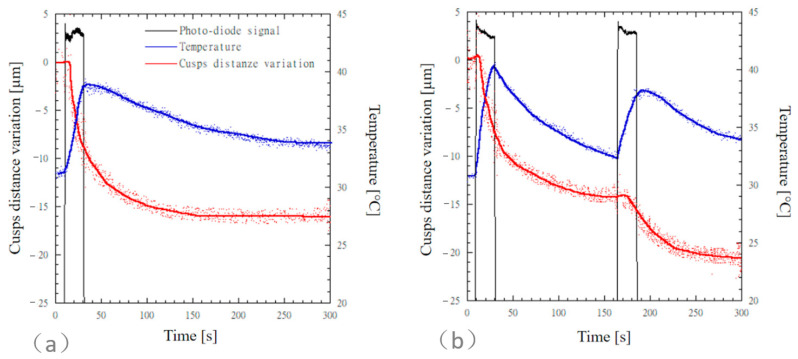
Simultaneous time measurements of cuspal distance variation, temperature, and photodiode signal for MOD cavities restored with SonicFill 2. The photodiode signal is reported on an arbitrary scale and it was used to detect the light curing and the dark reaction phases. MOD cavities restored according to (**a**) the bulk-fill technique and (**b**) the incremental technique.

Simultaneous time measurements of cuspal distance variation, temperature, and photodiode signal for MOD cavities restored with SonicFill 2 are reported in Figure 3. The photodiode signal is reported on an arbitrary scale, and it was used to detect the light curing and the dark reaction phases. For both the bulk-fill (Figure 3a) and the incremental (Figure 3b) techniques, a delay was observed in the cuspal distance variation before a steep contraction occurred.

A steep cuspal distance variation was observed for both the bulk-fill and the incremental techniques during the light curing phase, and cuspal distance variation still occurred at a lower steepness through the dark reaction phase (Figure 3). For the incremental technique, a further steep cuspal distance variation was observed as the second composite layer underwent polymerization. Mean values of cuspal distance variation of MOD cavities restored with SonicFill 2 were computed after 300 s and 3600 s (Table 4).

The cuspal distance variation measured for teeth restored through the incremental technique (20.2 ± 5.5 µm) was higher than that measured for teeth restored through the bulk-fill technique (16.3 ± 5.6 µm); however, the difference between the means was not statistically significant (*p* = 0.946). The temperature increases observed for SFLB and SFLI were 8.2 ± 1.6 °C and 9.5 ± 1.4 °C, respectively.

**Figure 4:**
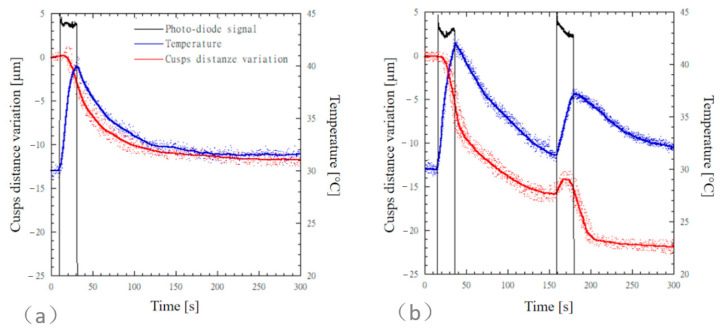
Simultaneous time measurements of cuspal distance variation, temperature, and photodiode signal for MOD cavities restored with SDR flow+. The photodiode signal is reported on an arbitrary scale and it was used to detect the light curing and the dark reaction phases. MOD cavities restored according to (**a**) the bulk-fill technique and (**b**) the incremental technique.

Simultaneous time measurements of cuspal distance variation, temperature, and photodiode signal for MOD cavities restored with SDR flow+ are reported in Figure 4. The photodiode signal is reported on an arbitrary scale and it was used to detect the light curing and the dark reaction phases. For both the bulk-fill (Figure 4a) and the incremental (Figure 4b) techniques, a delay was observed in the cuspal distance variation before a steep contraction occurred.

A steep cuspal distance variation was observed for both the bulk-fill and the incremental techniques during the light curing phase, and cuspal distance variation still occurred at a lower steepness through the dark reaction phase (Figure 4). For the incremental technique, a further steep cuspal distance variation was observed as the second composite layer underwent polymerization. Table 4 reports the mean values and standard deviation of cuspal distance variation of MOD cavities restored with SDR flow+ computed after 300 s and 3600 s. The cuspal distance variation measured for teeth restored through the incremental technique (21.8 ± 1.9 µm) was higher than that measured for teeth restored through the bulk-fill technique (11.8 ± 3 µm); however, the difference between the means was not statistically significant (*p* = 0.12). The temperature increases observed for SDRB and SDRI were 9.5 ± 1.7 °C and 11.9 ± 1.7 °C, respectively.

## 4. Discussion

Bulk-fill composites are particularly used in deep cavities, as they reduce the number of steps required for restoration. Bulk-fill composites are not significantly different from conventional composites in terms of microleakage [34]. Two types of bulk-fill materials (SDR flow plus and SonicFill 2) were used in our bulk-fill groups. SDR has more of an organic matrix than SonicFill (Table 1).

The mean shrinkage values (Figure 2) at 3600 s for SDR and SFL were 0.925 ± 0.041 µm/µm% and 0.523 ± 0.029 µm/µm%, respectively. Of course, the higher shrinkage of SDR was directly related to the amount of the polymeric matrix. It is worth noting that, at 3600 s, both the bulk-fill composites showed a positive steepness for the shrinkage profile (Figure 2), thus suggesting that shrinkage continued to increase. By using the equation reported by Garcia et al. [21], mean values of volumetric shrinkage of SDR and SFL were calculated as 2.75% and 1.56%, respectively. These volumetric shrinkage values are consistent but slightly lower than those measured for SDR and SFL with a linometer [21,22]. Instead, the volumetric shrinkage of 1.56% computed for SFL is consistent with that measured with a 3D camera imaging system [19]. Differences between our results and those reported in the literature may depend on several factors such as the technique adopted to measure shrinkage, the light curing energy provided for polymerization, and the time point at which shrinkage is measured. Shrinkage of light-cured restorative materials is a very complex phenomenon as the contraction differs along the three-dimensional directions, thus leading to an anisotropic shrinkage [35,36]. We measured shrinkage in the direction orthogonal to the light front through a strain gauge-based extensometer (Figure 1a) as this configuration better represents, in vitro, deformation occurring on the lingual and vestibular cusps of the restored MOD cavity (Figure 1c). Shrinkage measured in the direction orthogonal to the light front is lower than that measured in the axial direction of the light front through the linometer or the bonded disc method [36], as shrinkage of deep composite layers, undergoing polymerization kinetics lower than the surface layer [37,38], provide a significant contribution to the contraction measured in the axial direction.

Compressive properties of RBCs are of great importance as the stress due to mastication, acting on restored teeth, is mainly of compressive nature. The compressive strength and Young’s modulus in compression measured for SFL, significantly higher (*p* < 0.05) than those observed for SDR (Table 3), were directly ascribed to the amount of the filler reinforcement phase (Table 1). It is worth noting that, similarly to shrinkage, compressive properties also largely depended on the time point at which properties were measured. For both the investigated bulk-fill composites, significantly higher strength and stiffness (*p* < 0.05) were observed after 72 h (Table 3). The compressive strength values measured for SDR and SFL are consistent with values reported in the literature [24,25]. At both time points, SFL showed a compressive strength and Young’s modulus significantly higher (*p* < 0.05) than those recorded for SDR. Instead, at both time points, the strain to failure of SFL was significantly lower (*p* < 0.05) than that measured for SDR. The difference in mechanical properties observed for the two bulk-fill composites suggests that SFL is stiffer and more brittle than SDR, while SDR is more compliant than SFL.

Although several studies were developed regarding in vitro experimental cuspal deflection when conventional RBCs are used to restore MOD cavities of premolars [6,27,28,29,30,31,32], little is known on cuspal deflection when bulk-fill composites are used for restoring MOD cavities of premolars according to the incremental or the bulk-fill techniques. Kim et al. [33] recently evaluated cuspal deflection of aluminum teeth [6] according to a variety of conventional and bulk-fill composites considering both the incremental and the bulk-fill techniques, and a reduction in cuspal deflection from bulk to incremental layering was observed. Similarly to shrinkage (Figure 2) and compressive properties (Table 3), cuspal distance variation (Table 4) also depended on the time point at which measurements were taken. For each type of bulk-fill composite and layering technique, cuspal distance variation values at 3600 s were higher than those measured at 300 s. According to the bulk-fill technique, mean values of cuspal distance variation measured at 3600 s for SFLB and SDRB were 22.9 µm and 20.8 µm, respectively. These values were between the mean values measured for the same bulk-fill composite considering aluminum rectangular cusps having 1 mm and 2 mm thickness [33]. Cuspal deflection depends on bending stiffness; thus, it directly depends on the product between the second moment of area and the Young’s modulus. The mean width value of our premolar cusps (Table 2) was 6.47 mm, significantly lower than that of the aluminum cusps (8 mm) adopted by Kim et al. Moreover, the shape of the dental cuspal cross-section is semielliptical; hence, the second moment of area is lower than that of rectangular cross-section having similar dimensions. Additionally, the Young’s modulus of aluminum is about thrice that of dentine. For all these reasons, even if the mean cuspal thickness of our premolar cusps was 3.03 mm (Table 2), the bending stiffness of our premolar cusps was much lower than the bending stiffness of the aluminum teeth having similar thickness. Therefore, cuspal distance variations measured at 3600 s (Table 4) are consistent with the deflection of aluminum cusps measured by Kim et al. On the other hand, the mean value of cuspal distance variation measured for SFL (22.9 µm) is consistent with the mean value (24.3 µm) recorded for MOD restoration of human premolars by Nguyen et al. [39].

For SFL composite, no significant difference in cuspal distance variation was observed between the bulk-fill and the incremental layering techniques (*p* = 0.89). For the SDR composite, no significant difference in cuspal deflection was observed for the bulk-fill or the incremental layering techniques (*p* = 0.43).

Although a lower cuspal distance variation would be expected for SFL, as shrinkage values of SFL were significantly lower (*p* < 0.05) than those of SDR (Figure 2), no significant difference in cuspal deflection between SFLB and SDRB was observed (*p* = 0.99). Similarly, no significant difference in cuspal deflection between SFLI and SDRI was observed (*p* = 1.00). The Young’s modulus of SDR is significantly lower than that of SFL (Table 3); thus, it promotes elastic deformation of the material. SDR is more compliant than SFL; thus, it reduces the amount of stress on the cavity wall as the composite shrinks. Instead, the SFL composite generates high stress at cavity walls because of the higher stiffness, but this stress is compensated for by the lower shrinkage of this composite. Therefore, dental cuspal deflection for MOD cavities depends on both shrinkage and mechanical properties. High-viscosity composites (e.g., SFL), characterized by low shrinkage and high stiffness, produce similar cuspal deflection to low-viscosity composites (e.g., SDR), characterized by high shrinkage and low stiffness.

Concerning the bulk-fill technique (Figure 3a or Figure 4a), mean values of temperature rise ranged from 8.2 °C to 9.5 °C, and higher temperature levels were recorded for the flowable composite SDR. However, the difference between the mean values recorded for the different composites was not significant (*p* = 0.92). Similarly, for the incremental layering technique (Figure 3b or Figure 4b), temperature rise values recorded through the first increment ranged from 9.5 °C to 11.9 °C, and higher temperature levels were recorded for the flowable composite SDR. However, the difference between the mean values recorded for the different composites was not significant (*p* = 0.99). Karacan and Ozyurt recorded similar temperature increase values with thermocouples placed 1 mm below the MOD cavity using a high-viscosity bulk-fill composite [40]. The temperature rise occurring in the core of SDR composite, higher than 20 °C, was measured using different techniques [7,17,41]. These temperature levels would be detrimental for the pulp tissue. Fortunately, dentin acts as a thermal insulator system since the thermal conductivity of dentin [42] effectively reduces temperature rise occurring in the pulp. Therefore, for both SDR and SFL composites, the light curing modality of 1000 mW/cm^2^ for 20 s can be considered thermally safe if an appropriate thickness of occlusal dentin is preserved [40,43,44,45].

Since no significant difference was observed for both cuspal distance variation and temperature rise, according to the different restorative materials and layering techniques, the null hypothesis was not rejected. The stress distribution occurring at the interface between bulk-fill composites and dental cusps is of paramount importance for the long-term stability of the restoration. Cuspal distance variation measured within this investigation can be used to calibrate finite element models of MOD cavities restored through bulk-fill composites. Future developments will consider experimental mechanical testing in conjunction with finite element modeling. Moreover, different cavity preparations will be considered to evaluate the effect of cavity morphology on dental cusp deformation and on stress distribution.

## 5. Conclusions

On the basis of the reported results, the following conclusions can be drawn:

- Shrinkage of SDR was significantly higher (*p* < 0.05) than that of SonicFill, while the strength and the Young’s modulus of Sonic Fill were significantly higher (*p* < 0.05) than those of SDR.
- For both SDR and SonicFill composites, no significant difference (*p* = 0.95 and *p* = 0.12, respectively) was observed for cuspal distance variation according to MOD cavities restored through the bulk-fill or the incremental layering techniques.
- Although a lower cuspal distance variation would be expected for Sonic Fill, as shrinkage values of SonicFill were lower than SDR, no significant difference in cuspal deflection between SonicFill and SDR was observed according to both the bulk fill and the incremental layering techniques. This result can be ascribed to the Young’s modulus, suggesting that SonicFill is stiffer than SDR, while SDR is more compliant than SonicFill.
- Temperature rise levels were below 11.9 °C; however, no significant difference in the mean values of temperature rise was observed between the bulk-fill composites and the layering techniques.
- The curing modality (1000 mW/cm^2^ for 20 s) can be considered thermally safe for the pulp tissue if the thickness of the occlusal dentin is not lower than 1 mm. However, for curing flowable composites applied in very deep cavities, it is recommended to use lower light intensity in conjunction with an increased exposure time.

## Figures and Tables

**Table 1:** Composite material composition and restoration techniques.

Material	Manufacturer	Composition	Restorative Technique	Acronym *
**SonicFill 2**	Kerr, USA	Matrix: Bis-GMA, TEGDMA, EBPDMA Filler 83.5 wt.%: SiO_2_, glass, oxide	Bulk	SFLB
			Incremental	SFLI
**SDR flow+**	Sirona Dentsply, USA	Matrix: UDMA, TEGDMA, EBPDMA Filler 68 wt.%: Ba–Al–F–B–Si glass and St–Al–F–Si glass	Bulk	SDRB
			Incremental	SDRI

* SFLB: SonicFill 2 restored through the bulk-fill technique; SFLI: SonicFill 2 restored through the incremental technique; SDRB: Smart Dentin Replacement restored through the bulk-fill technique; SDRI: Smart Dentin Replacement restored through the incremental technique.

**Table 2:** Teeth cusps and restorations of mesio-occlusal–distal (MOD) cavity mean dimensions expressed in mm. Numbers in brackets represent the standard deviation.

	MOD Thickness	MOD Height	MOD Width	Vestibular Cusp Height	Lingual Cusp Height	Vestibular Cusp Width	Lingual Cusp Width	Vestibular Cusp Thickness	Lingual Cusp Thickness
**SFLB**	3.05 (0.65)	4.07 (0.20)	6.67 (0.28)	6.48 (0.81)	5.88 (1.02)	7.1 (0.31)	6.21 (0.31)	3.16 (0.10)	3.06 (0.35)
**SFLI**	2.85 (0.57)	3.96 (0.43)	6.51 (0.39)	6.67 (0.72)	5.59 (0.86)	6.88 (0.25)	6.14 (0.64)	3.41 (0.31)	3.35 (0.42)
**SDRB**	2.96 (0.48)	3.83 (0.56)	6.44 (0.21)	6.05 (0.53)	5.57 (1.1)	6.64 (0.50)	6.20 (0.08)	3.15 (0.33)	2.71 (0.40)
**SDRI**	3.29 (0.17)	4.01 (0.61)	6.60 (0.12)	5.97 (0.59)	5.36 (0.82)	6.9 (0.11)	6.26 (0.13)	3.02 (0.42)	2.90 (0.39)

**Table 3:** Mechanical properties measured in compression for the SDR and SFL bulk-fill composites. σ, ε, and E represent the compressive strength, the strain to failure, and the Young’s modulus, respectively. Numbers in brackets represent the standard deviation.

Bulk-Fill Composite	Time t0			Time 72 h		
	σ (MPa)	ε (%)	E (GPa)	σ (MPa)	ε (%)	E (GPa)
**SDR**	234 (13)	19.4 (0.3)	1.7 (0.1)	277 (10)	16.7 (0.3)	3.1 (0.1)
**SFL**	297 (18)	6.4 (0.4)	5.1 (0.2)	329 (36)	5.9 (0.5)	8.5 (0.4)

**Table 4:** Cuspal distance variation at 300 s and at 3600 s.

	300 s		3600 s	
Material	Mean Value (SD) (μm)	*p*-Value	Mean Value (SD) (μm)	*p*-Value
**SFLB**	16.3 (5.6)	0.95	22.9 (9.2)	0.89
**SFLI**	20.2 (5.5)		27.5 (7.2)	
**SDRB**	11.8 (3.0)	0.12	20.8 (3.5)	0.43
**SDRI**	21.8 (1.9)		28.3 (5.0)	
